# Congenital hypogonadotropic hypogonadism and constitutional delay of growth and puberty have distinct genetic architectures

**DOI:** 10.1530/EJE-17-0568

**Published:** 2018-02-01

**Authors:** Daniele Cassatella, Sasha R Howard, James S Acierno, Cheng Xu, Georgios E Papadakis, Federico A Santoni, Andrew A Dwyer, Sara Santini, Gerasimos P Sykiotis, Caroline Chambion, Jenny Meylan, Laura Marino, Lucie Favre, Jiankang Li, Xuanzhu Liu, Jianguo Zhang, Pierre-Marc Bouloux, Christian De Geyter, Anne De Paepe, Waljit S Dhillo, Jean-Marc Ferrara, Michael Hauschild, Mariarosaria Lang-Muritano, Johannes R Lemke, Christa Flück, Attila Nemeth, Franziska Phan-Hug, Duarte Pignatelli, Vera Popovic, Sandra Pekic, Richard Quinton, Gabor Szinnai, Dagmar l’Allemand, Daniel Konrad, Saba Sharif, Özlem Turhan Iyidir, Brian J Stevenson, Huanming Yang, Leo Dunkel, Nelly Pitteloud

**Affiliations:** 1 Service of EndocrinologyDiabetology and Metabolism, Lausanne University Hospital, Lausanne, Switzerland; 2 Faculty of Biology and MedicineUniversity of Lausanne, Lausanne, Switzerland; 3 Centre for EndocrinologyWilliam Harvey Research Institute, Barts and the London School of Medicine and Dentistry, Queen Mary University of London, London, UK; 4 BGI-ShenzhenShenzhen, China; 5 Shenzhen Key Laboratory of NeurogenomicsBGI-Shenzhen, Shenzhen, China; 6 Centre for Neuroendocrinology (Royal Free Campus)University College London, London, UK; 7 University Hospital BaselClinic of Gynecological Endocrinology and Reproductive Medicine, Basel, Switzerland; 8 Center for Medical GeneticsGhent University Hospital, Ghent, Belgium; 9 Section of Investigative MedicineImperial College London, Hammersmith Hospital, London, UK; 10 Rue du Curtil-MailletYverdon-les-Bains, Switzerland; 11 Division of Pediatric Endocrinology and Diabetology and Children’s Research CentreUniversity Children’s Hospital, Zurich, Switzerland; 12 Institute of Human GeneticsUniversity of Leipzig Hospitals and Clinics, Leipzig, Germany; 13 Pediatric Endocrinology and DiabetologyDepartment of Clinical Research, University Children’s Hospital Bern, Bern, Switzerland; 14 St. John’s HospitalBudapest, Hungary; 15 Serviço de EndocrinologiaDiabetes e Metabolismo, Hospital de São João e Faculdade de Medicina do Porto, Porto, Portugal; 16 School of MedicineUniversity of Belgrade, Belgrade, Serbia; 17 Clinic for EndocrinologyDiabetes and Diseases of Metabolism, University Clinical Center, Belgrade, Serbia; 18 Department of EndocrinologyInstitute for Human Genetics, University of Newcastle-upon-Tyne, Newcastle-upon-Tyne, UK; 19 University of Basel Chidren's HospitalBasel, Switzerland; 20 Department of EndocrinologyChildren’s Hospital of Eastern Switzerland, St Gallen, Switzerland; 21 Clinical Genetics UnitBirmingham Women’s Hospital, Birmingham, UK; 22 Department of Endocrinology and MetabolismGazi University Faculty of Medicine, Ankara, Turkey; 23 SIB Swiss Institute of BioinformaticsLausanne, Switzerland; 24 James D. Watson Institute of Genome SciencesHangzhou, China

## Abstract

**Objective:**

Congenital hypogonadotropic hypogonadism (CHH) and constitutional delay of growth and puberty (CDGP) represent rare and common forms of GnRH deficiency, respectively. Both CDGP and CHH present with delayed puberty, and the distinction between these two entities during early adolescence is challenging. More than 30 genes have been implicated in CHH, while the genetic basis of CDGP is poorly understood.

**Design:**

We characterized and compared the genetic architectures of CHH and CDGP, to test the hypothesis of a shared genetic basis between these disorders.

**Methods:**

Exome sequencing data were used to identify rare variants in known genes in CHH (*n* = 116), CDGP (*n* = 72) and control cohorts (*n* = 36 874 ExAC and *n* = 405 CoLaus).

**Results:**

Mutations in at least one CHH gene were found in 51% of CHH probands, which is significantly higher than in CDGP (7%, *P* = 7.6 × 10^−11^) or controls (18%, *P* = 5.5 × 10^−12^). Similarly, oligogenicity (defined as mutations in more than one gene) was common in CHH patients (15%) relative to CDGP (1.4%, *P* = 0.002) and controls (2%, *P* = 6.4 × 10^−7^).

**Conclusions:**

Our data suggest that CDGP and CHH have distinct genetic profiles, and this finding may facilitate the differential diagnosis in patients presenting with delayed puberty.

## Introduction

Congenital hypogonadotropic hypogonadism (CHH (MIM: 146110)) is a rare disorder affecting approximately 1 in 4000 births (1). It is caused by GnRH deficiency, and subsequently results in altered activation of the hypothalamic–pituitary–gonadal (HPG) axis that controls sexual maturation and fertility. Clinically, CHH presents as absent/incomplete puberty and infertility. It is characterized by isolated low sex steroids in the setting of low (or inappropriately normal) serum gonadotropins in the absence of other hypothalamo-pituitary defects. Clinically, CHH is a heterogeneous disorder. In the presence of anosmia (the inability to smell) in approximately 50% of CHH probands, the condition is termed Kallmann syndrome (KS (MIM: 308700)). Other associated phenotypes such as hearing loss, synkinesia, renal agenesis, ataxia and cleft lip/palate are also observed with variable frequency (2). Interestingly, a higher than expected proportion (10%) of family members of CHH probands report a history of delayed puberty (3). Moreover, reversal of hypogonadotropic hypogonadism in CHH patients after discontinuing hormone therapy also points to a clinical overlap between the two entities (4). Therefore, congenital delay of growth and puberty (CDGP), also termed self-limited delayed puberty, and CHH appear to be part of the same clinical spectrum – one being classically described as transient (CDGP) and the other as permanent (CHH) (3). In contrast to CHH, CDGP is a common disease, observed in 2–2.5% of the population (5).

Since the initial genetic report implicating *KAL1* (now *ANOS1*) (6, 7), the genetics of CHH has been widely studied. Similar to its diverse clinical presentation, the genetic architecture of CHH is also heterogeneous, with several modes of inheritance having been described including autosomal dominant, autosomal recessive, X-linked and *de novo*. Mutations in more than 30 genes have been shown to cause CHH (2); however, they only account for approximately 35% of cases (8). Defects in genes involved in GnRH neuron development and olfactory system usually result in KS, whereas mutations in genes involved in GnRH secretion or homeostasis result in normosmic CHH (nCHH). Interestingly, clinical overlap between KS and nCHH has been reported, with a disease spectrum rather than a binary classification for normosmic and anosmic (9). In parallel, genetic overlap between KS and nCHH also exists, with several genes mutated in both subgroups (2).

Although long thought to be a monogenic disorder, frequent observations of incomplete penetrance and variable expressivity within and across families suggested this model was insufficient to fully explain the observed phenotypes in CHH. Indeed, previous work by our team and others has shown that oligogenic inheritance (i.e. more than one gene mutated in the same individual) can at least partially explain some of these phenomena (8, 10). Synergistic effects between CHH genes have been also described *in vitro* (e.g. *FGF8*/*FGFR1*) (11) and *in vivo* (e.g. *KISS1*/*KISS1R*) (12). Oligogenicity has been proposed in heterogeneous genetic disorders such as Bardet–Biedl syndrome (BBS) (13) and retinitis pigmentosa (14). In addition, oligogenicity is also proposed for other endocrine diseases such as premature ovarian failure (15, 16) with the constellation of more than one gene mutated.

Pubertal timing is a highly heritable trait as up to 50–80% of the variance is explained by genetic factors (17). Consistently, CDGP runs in families with complex inheritance pattern (18), but in contrast to CHH, little is known about the genetics of CDGP. A recent study identified mutations with low frequencies (MAF <2.5%) in *IGSF10* in 13% of CDGP probands. IGSF10 is a large protein that is part of the immunoglobulin superfamily and appears to have a developmental role in GnRH neuron migration (19). In addition, genome-wide association studies (GWAS) evaluating common and rare variants in the timing of puberty identified significant associations with hundreds of loci, including regions near or within *ANOS1*,* TACR3*, *LEPR* and *PCSK1* – four known CHH genes. Taken together, these loci account for <3% of the variance in age of puberty onset (20, 21). In view of the possible overlap between the pathophysiology of delayed puberty and conditions of GnRH deficiency, few studies have searched for mutations in CHH genes in CDGP cohorts. A homozygous partial loss-of-function mutation in *GNRHR* was found in two brothers, one with CDGP and one with CHH (22). Of 50 CDGP patients investigated for mutations in *TAC3* and *TACR3*, only one mutation in a single patient was found in the latter gene (23). However, a recent study screening 21 CHH genes in a CDGP cohort (*n* = 56) found potentially pathogenic mutations in 14% of patients (3). Recently, low frequency (MAF <2.5%) potentially pathogenic variants in *IGSF10* were found in 10% of CHH patients (19), suggesting the hypothesis of a partial genetic overlap between CHH and CDGP.

Currently, the differential diagnosis between CHH and CDGP at early adolescence remains challenging, as both conditions present with isolated delay in puberty. Further, there are no specific biochemical markers to accurately differentiate these two disorders (24). In the current study, we explored the genetic architecture of both CHH and CDGP and to investigate whether genetic testing could assist in the differential diagnosis. We also characterized the genetic overlap between KS and nCHH using a comprehensive screening of all CHH genes and defined the mutational spectrum of CHH genes in the control population.

## Subjects and methods

### Patient and control cohorts

The study cohort includes 116 CHH probands of European descent (*n* = 61 KS, *n* = 55 nCHH) with a 2:1 male-to-female ratio consistent with previous reports of male predominance (1). The diagnosis of CHH was determined by (1) absent or partial puberty by 17 years (25), (2) low/normal serum gonadotropin levels in the setting of low serum testosterone/estradiol levels, (3) otherwise normal anterior pituitary function and (4) normal imaging of the hypothalamic–pituitary area (25). Olfaction was assessed by self-report and/or formal testing (9) using the UPSIT or Sniffin’ Stick tests. When possible, family members were recruited for clinical and genetic studies.

The delayed puberty cohort consists of 72 unrelated probands with CDGP of primarily Finnish European origin and has been previously described in detail (26). All patients met the diagnostic criteria for CDGP, defined as (1) onset of Tanner genital stage II two SDs later than population average (i.e. in boys testicular volume >3 mL after 13.5 years of age and in girls Tanner breast stage II after 13.0 years of age) (27). Medical history, clinical examination and routine laboratory tests were performed to exclude chronic illnesses, and the diagnosis of CHH was ruled out by spontaneous pubertal development at follow-up. All patients were followed until near-full pubertal development was attained (i.e., Tanner stage 4).

Ethnically matched controls (non-Finnish European (NFE) and Finnish European (FIN)) from the Exome Aggregation Consortium (ExAC) (28) were used for individual variant and gene mutation frequencies. Oligogenicity was assessed using the ‘Cohorte Lausannoise’ (CoLaus) control population, consisting of 405 participants of mixed European origin, phenotyped as described by Firmann and coworkers (29). This population-based cohort was assembled as part of a cardiovascular risk study, and therefore, has a typical distribution of pubertal age relative to the general population. The ages of the cohort participants are 35–75 years old (mean 51 ± 11 years).

### DNA extraction and sequencing

DNA was extracted from peripheral blood leukocytes using the PureGene kit (QIAGEN). Exome sequencing was performed on CHH and CDGP cohorts using the SureSelect V2 or V5 probes (Agilent) or the Nimblegen SeqCap EZ Exome V2 (Roche) and sequenced on the HiSeq 2000 platform (Illumina, San Diego, CA, USA) at either BGI (BGI, Shenzen, PRC) or Otogenetics (Otogenetics Corp., Atlanta, GA, USA). Exome sequencing on CoLaus DNA was performed at the Wellcome Trust Sanger Institute (WTSI) as part of a partnership between the Institute, the CoLaus principal investigators and the Quantitative Sciences department of GlaxoSmithKline (GSK, Brentford, UK).

### Definition of genes to be screened

‘CHH genes’ are those which met the following criteria: (1) identified as CHH genes in Boehm and coworkers (2), (2) had publications demonstrating loss-of-function variants, (3) had been demonstrated to be expressed in organs/tissues relevant for GnRH biology and (4) covered by the exome capture probes. Twenty-four genes met these criteria: *ANOS1*, *SEMA3A*, *FGF8*, *FGF17*, *SOX10*, *IL17RD*, *AXL*, *FGFR1*, *CHD7*, *HS6ST1*, *PCSK1*, *LEP*, *LEPR*, *FEZF1*, *NSMF*, *PROKR2*, *WDR11*, *PROK2*, *GNRH1*, *GNRHR*, *KISS1*, *KISS1R*, *TAC3* and *TACR3*. In addition, we screened the *IGSF10* gene, recently implicated in CDGP and CHH (19).

### Bioinformatics analysis and downstream variants filtering

Exome sequences from CHH probands, CDGP probands and CoLaus controls were analyzed following the Genome Analysis Toolkit (GATK) Best Practices (30). The computations were performed at the Vital-IT Center for High-Performance Computing of the Swiss Institute of Bioinformatics. Variants called with a genotype quality (GQ) <50 were excluded. The complete set of CHH gene variants from the ExAC database was downloaded from the ftp site (ftp://ftp.broadinstitute.org/pub/ExAC_release/release0.3). Annotation was performed using SnpEff (31), version 4.0. Intronic variants within ±6 bp of exonic boundaries and predicted to affect splicing by MaxEnt (32) with a wild-type vs mutated site change of ±20% were retained, as well as inframe/frameshift indels, stop gain, and missense variants. Protein-truncating variants (PTVs) were defined as frameshift, stop gain and splice variants (28).

For the purpose of this study, we define as mutations (1) rare (MAF <1%) PTVs, (2) rare missense variants predicted to be damaging to protein function by at least one *in silico* algorithm (SIFT (33) or PolyPhen-2 (34)) and (3) loss-of-function variants based on *in vitro* studies, regardless of *in silico* predictions.

### Statistical analyses

Statistics for individual and oligogenic variants in cases vs controls were performed using a two-tailed Fisher’s exact test. Gene-based allele frequencies in ExAC were calculated dividing the sum of alternate allele counts in ethnically matched samples with the average of alleles inspected. Gene-collapsed rare variant association (RVA) tests in cases vs controls were calculated using mutated allele frequencies in a two-tailed Fisher’s exact test. Statistical significance in gene-based RVA tests was defined using Bonferroni correction, dividing nominal significance (0.05) with the number of tests performed (i.e. genes analyzed, *n* = 25); hence, the cutoff to determine significance was set at *P* = 0.002.

### Ethics approval and consent to participate

This study was approved by the ethics committee of the University of Lausanne. All participants provided written informed consent prior to study participation. The study protocol was approved by the Ethics Committee for Paediatrics, Adolescent Medicine and Psychiatry, Hospital District of Helsinki and Uusimaa (and extended to encompass Kuopio, Tampere and Turku University Hospitals) (570/E7/2003). UK ethical approval was granted by the London-Chelsea NRES committee (13/LO/0257). The study was conducted in accordance with the guidelines of The Declaration of Helsinki.

## Results

### CHH genes are mutated in 51% of CHH probands but only in 7% in CDGP probands

**Figure 1:**
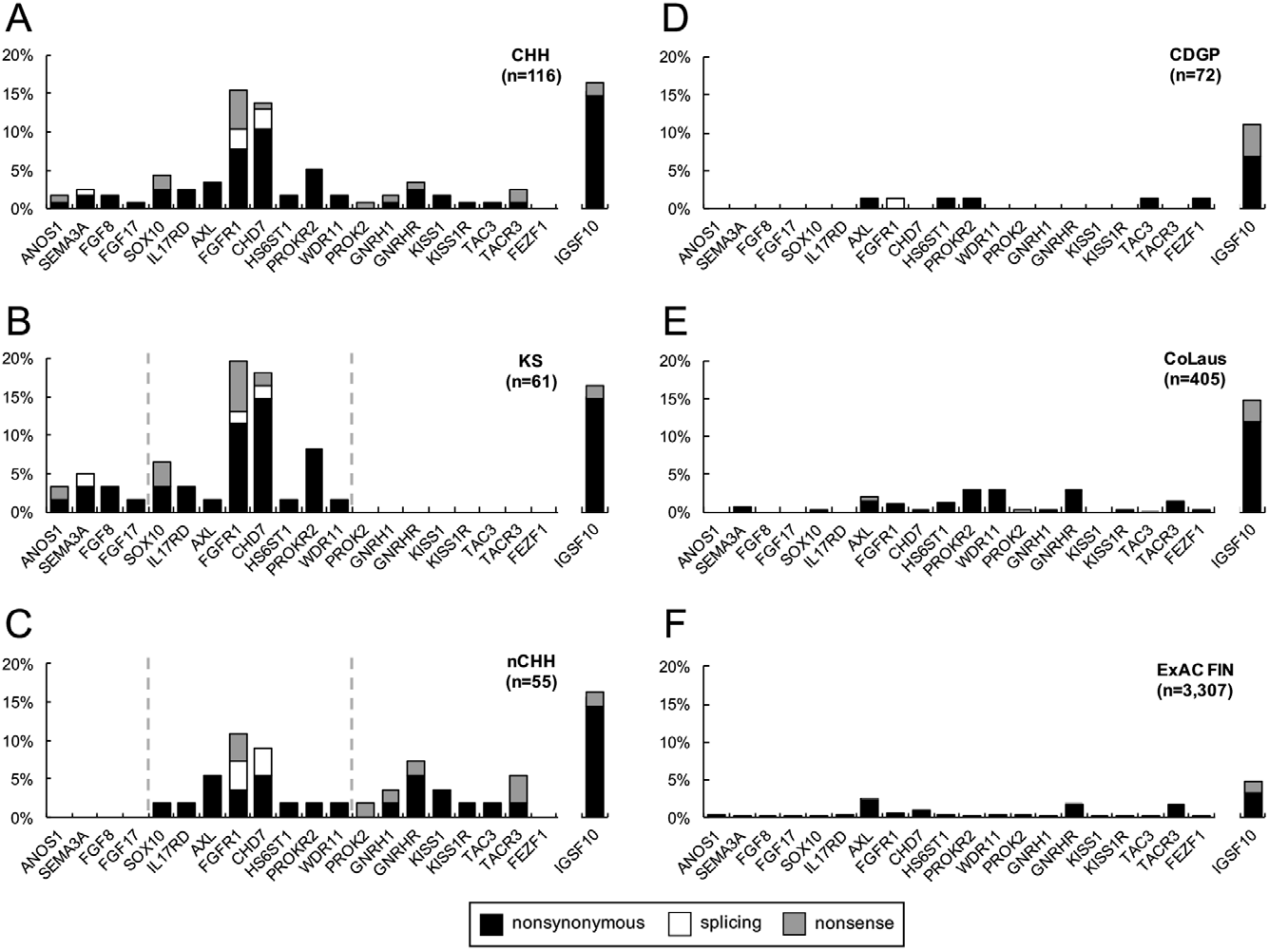
KS and nCHH display both shared and specific genetic patterns, and CDGP is not characterized by genetic overlap with CHH. Histograms showing CHH genes and *IGSF10* mutational prevalence in (A) CHH, (B) KS, (C) nCHH, (D) CDGP, (E) CoLaus, and (F) ExAC Finnish (FIN) cohorts. The prevalence of probands with variants in each gene are noted in black for nonsynonymous (i.e. missense and inframe InDels), white for splicing, and gray for nonsense (i.e. frameshift and stop gained) variants.

**Figure 2 fig2:**
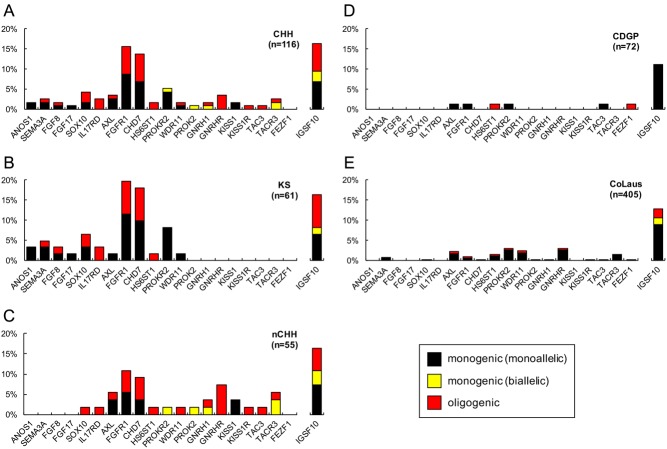
The majority of CHH genes are inherited in a oligogenic fashion in CHH probands, a trend not observed in CDGP probands and CoLaus controls. Histograms showing CHH genes mutational prevalence in (A) CHH, (B) KS, (C) nCHH, (D) CDGP and (E) CoLaus screened individuals. Each bar contains the frequency of each gene inheritance: monoallelic (gray), biallelic (yellow) or oligogenic (red).

Exome sequencing was performed on 116 CHH probands, and 59 (51%) harbored mutations in 20 of the 25 genes evaluated (Fig. 1A and Supplementary Table 1, see section on supplementary data given at the end of this article). No mutations were identified in *NSMF*, *FEZF1*, *PCSK1*, *LEP* and *LEPR*. Nearly two-thirds of familial CHH probands carried mutations in CHH genes (27/44, 61%), while the frequency in sporadic probands was lower (32/72, 44%) (Supplementary Fig. 2).[Fig fig2]

*FGFR1* and *CHD7* were the most frequently mutated genes in CHH probands (Fig. 1A), and both were statistically enriched for mutations compared to ExAC NFE controls (Table 1 and Supplementary Fig. 1). All of the identified *FGFR1* and *CHD7* mutations were present in a heterozygous state (Supplementary Table 2). In addition, a significant enrichment of mutations was observed for *SOX10*, with a prevalence of 4% in CHH patients (Table 1 and Supplementary Table 1).

**Table 1:** CHH known genes mutated allele frequencies in CHH, KS, nCHH, CDGP probands and CoLaus, 1000 Genomes and ExAC European controls.

	Phenotype reported	CHH	RVA test			KS	RVA test			nCHH	RVA test			% mutated alleles				
		% mutated alleles	vs CoLaus	vs 1000 Genomes EUR	vs ExAC NFE	% mutated alleles	vs CoLaus	vs 1000 Genomes EUR	vs ExAC NFE	% mutated alleles	vs CoLaus	vs 1000 Genomes EUR	vs ExAC NFE	CDGP	CoLaus	1000 Genomes EUR	ExAC FIN	ExAC NFE
*ANOS1*	KS	0.9	ns	ns	ns	1.6	ns	ns	ns	0.0	ns	ns	ns	0.0	0.0	0.5	0.22	0.64
*SEMA3A*	KS	1.3	ns	ns	ns	2.5	ns	ns	ns	0.0	ns	ns	ns	0.0	0.4	0.5	0.11	0.45
*FGF8*	KS, nCHH	0.9	ns	ns	ns	1.6	ns	ns	**0.002**	0.0	ns	ns	ns	0.0	0.0	0.0	0.02	0.06
*FGF17*	KS, nCHH	0.4	ns	ns	ns	0.8	ns	ns	ns	0.0	ns	ns	ns	0.0	0.0	0.0	0.15	0.02
*SOX10*	KS	2.2	ns	ns	**4.4E-06**	3.3	**2.8E-04**	ns	**7.7E-06**	0.9	ns	ns	ns	0.0	0.1	0.0	0.03	0.09
*IL17RD*	KS	1.3	ns	ns	ns	1.6	ns	ns	ns	0.9	ns	ns	ns	0.0	0.0	0.8	0.24	0.67
*AXL*	KS, nCHH	1.7	ns	ns	ns	2.5	ns	ns	ns	2.7	ns	ns	ns	0.7	1.0	1.0	1.27	1.59
*FGFR1*	KS, nCHH	7.8	**1.3E-08**	**3.8E-07**	**8.5E-14**	9.8	**1.8E-08**	**2.1E-07**	**6.6E-11**	5.5	ns	ns	**1.1E-04**	0.7	0.5	0.3	0.35	0.68
*CHD7*	KS, nCHH	6.9	**1.6E-07**	**2.9E-04**	**2.6E-05**	9.0	**1.4E-09**	**1.3E-04**	**4.2E-05**	4.5	**1.2E-04**	ns	ns	0.0	0.1	1.3	0.47	2.06
*HS6ST1*	KS, nCHH	0.9	ns	ns	ns	0.8	ns	ns	ns	0.9	ns	ns	ns	0.7	0.7	0.5	0.24	0.72
*PROKR2*	KS, nCHH	3.0	ns	ns	ns	4.1	ns	ns	ns	1.8	ns	ns	ns	0.7	1.5	1.0	0.14	1.24
*WDR11*	KS, nCHH	0.9	ns	ns	ns	0.8	ns	ns	ns	0.9	ns	ns	ns	0.0	1.5	0.8	0.20	1.22
*PROK2*	KS, nCHH	0.9	ns	ns	ns	0.0	ns	ns	ns	1.8	ns	ns	ns	0.0	0.1	0.0	0.23	0.08
*GNRH1*	nCHH	1.3	ns	ns	ns	0.0	ns	ns	ns	2.7	ns	ns	ns	0.0	0.1	0.0	0.02	0.23
*GNRHR*	nCHH	3.0	ns	ns	ns	0.0	ns	ns	ns	6.4	ns	**1.3E-04**	**5.7E-05**	0.0	1.5	0.3	0.92	0.88
*KISS1*	nCHH	0.9	ns	ns	ns	0.0	ns	ns	ns	1.8	ns	ns	**0.002**	0.0	0.0	0.0	0.03	0.06
*KI*	nCHH	0.4	ns	ns	ns	0.0	ns	ns	ns	0.9	ns	ns	ns	0.0	0.1	0.3	0.03	0.15
*TAC3*	nCHH	0.4	ns	ns	ns	0.0	ns	ns	ns	0.9	ns	ns	ns	0.7	0.1	0.0	0.02	0.04
*TACR3*	nCHH	2.2	ns	ns	ns	0.0	ns	ns	ns	4.5	ns	ns	**0.0017**	0.0	0.7	0.5	0.92	0.29

Rare variant association (RVA) test was performed via a two-sided Fisher’s exact test. Association with *P* ≤ 0.002 (in bold) were considered significant after Bonferroni correction.

CDGP, constitutional delay of growth and puberty; CHH, congenital hypogonadotropic hypogonadism; KS, Kallmann syndrome; nCHH, normosmic congenital hypogonadotropic hypogonadism; ns, not significant.

Exome sequencing identified 7% (*n* = 5) of CDGP probands harboring mutations in the known CHH genes, all of which are heterozygous (Fig. 1D and Supplementary Table 3). Thus, the genetic profile of the CDGP cohort more closely resembles the controls (both ExAC Finnish and non-Finnish controls) rather than CHH probands. Among the six identified mutations, there were five missense and one intronic change predicted to affect splicing. Three mutations were private compared to 3307 Finnish ExAC controls. Only one CDGP proband harbored two mutated genes (oligogenicity) (1.4%, *P* = 0.002 vs CHH), a similar rate as observed in controls ( and Supplementary Table 2). Clinically, this CDGP patient had spontaneous puberty at 14.3 years and achieved normal adult testicular volume and testosterone levels over the subsequent 2.4 years, thereby excluding a diagnosis of CHH.

**Figure 3:**
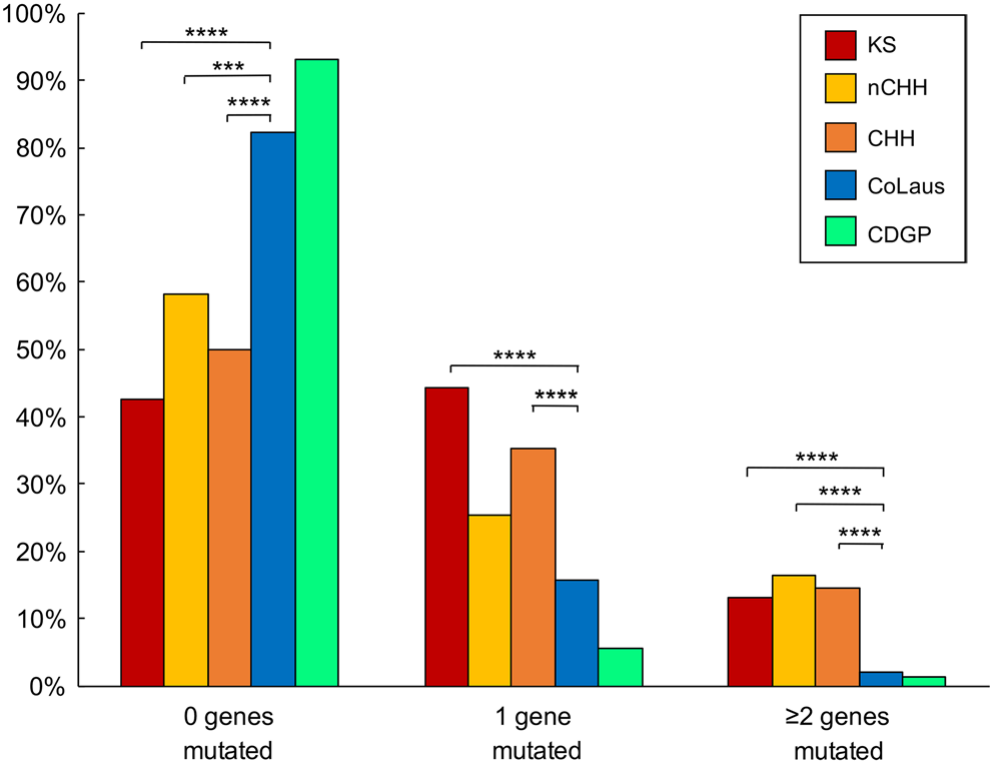
Oligogenicity is a common factor in CHH, and it is not found in CDGP. Histogram showing the frequency of KS (red), nCHH (yellow), CHH (orange), CoLaus (blue) and CDGP (green) individuals having no rare variants in CHH genes, one gene mutated or at least two genes mutated (oligogenicity). Differences between KS, nCHH and CHH vs CoLaus controls were analyzed via a two-sided Fisher’s exact test. *P* < 0.05 was considered significant. *P < 0.05; **P < 0.01; ***P < 0.001; ****P < 0.0001. Not significant differences among KS, nCHH and CHH vs CoLaus are not displayed.

### Prevalence of putative *IGSF10* mutations in CHH is similar to CDGP

We found a large number of CHH patients (19/116, 16.4%) harboring putative *IGSF10* mutations compared to CDGP (8/72, 11.1%) (Fig. 1A, B, C and D). Our data did not show enrichment for mutations in our cohorts as similar frequencies were seen in controls (Fig. 1A, B, C and D).

### KS and nCHH show both exclusive and shared genetic architectures

We examined the mutational spectrum relative to the two subgroups of CHH – KS (*n* = 61) and nCHH (*n* = 55). Among KS, *FGFR1* and *CHD7* were the most frequently mutated genes, and together with *SOX10* are significantly enriched when compared to controls (Fig. 1B and Table 1). This finding is even more robust when evaluating the KS subgroup alone. Similarly, *FGF8* showed a prevalence of 1.6% in KS; yet, this association was not evident in the CHH cohort as a whole. Mutations in *ANOS1*, *SEMA3A*,* FGF17* and* FGF8* were only found in KS.

Among normosmic probands (nCHH),* FGFR1* and *CHD7* were also the most frequently mutated genes. Mutations in *GNRHR* and *TACR3* were only found in nCHH (7% and 5%, respectively) (Fig. 1C). Further, *FGFR1*, *KISS1*, *GNRHR* and *TACR3* were significantly enriched in nCHH cases compared to ExAC NFE controls (Table 1).

In addition to *FGFR1* and *CHD7*, six other CHH genes (*SOX10*, *IL17RD*, *AXL*, *HS6ST1*, *PROKR2* and *WDR11*) were mutated in both KS and nCHH (Fig. 1B and C). This represents an increased genetic overlap in comparison to prior report (2). Overall, these results indicate both exclusive and shared genetic architectures for both KS and nCHH.

### nCHH probands are enriched with biallelic mutations

Biallelic mutations (i.e. homozygous or compound heterozygous changes in the same gene) were found exclusively in nCHH (6/55, 11%) and were not seen in KS (*P* = 0.01), CDGP (*P* = 0.006) or in CoLaus (*P* = 2.3 × 10^−6^) (Fig. 2). Furthermore, 4/15 (27%) genes mutated in nCHH (*GNRHR*, *GNRH1*, *PROKR2*, *PROK2*, *TACR3*) only exhibited biallelic mutations, consistent with their recessive mode of inheritance (Fig. 2C and Supplementary Table 1).

### Oligogenicity is a common factor in CHH patients

Oligogenicity was present in 17/116 (15%) of CHH probands (Fig. 3) – a higher frequency than previous reports of 2.5–7% (8, 10). A significantly lower rate of oligogenicity was observed in controls (CoLaus: 2%,* P* = 6.4 × 10^−7^).

Additionally, although monogenic inheritance was more frequent in KS (46%) compared to nCHH (25%, *P* = 0.03), CDGP (6%, *P* = 3.7 × 10^−8^) and CoLaus (16%, *P* = 4.6 × 10^−7^), similar frequencies of oligogenicity were identified in both KS (13%) and nCHH (16%) (Fig. 3 and Supplementary Table 3).

Among the 20 mutated genes identified in CHH patients, 84% (*n* = 16 genes) participated in oligogenicity (Fig. 2A). Of these 16 genes, mutations in *IL17RD*, *HS6ST1*, *KISS1R* and *TAC3* occurred exclusively in an oligogenic manner. *ANOS1*, *FGF17*,* KISS1* and *PROK2* were the only genes exclusively showing monogenic inheritance (Fig. 2A).

**Figure 4:**
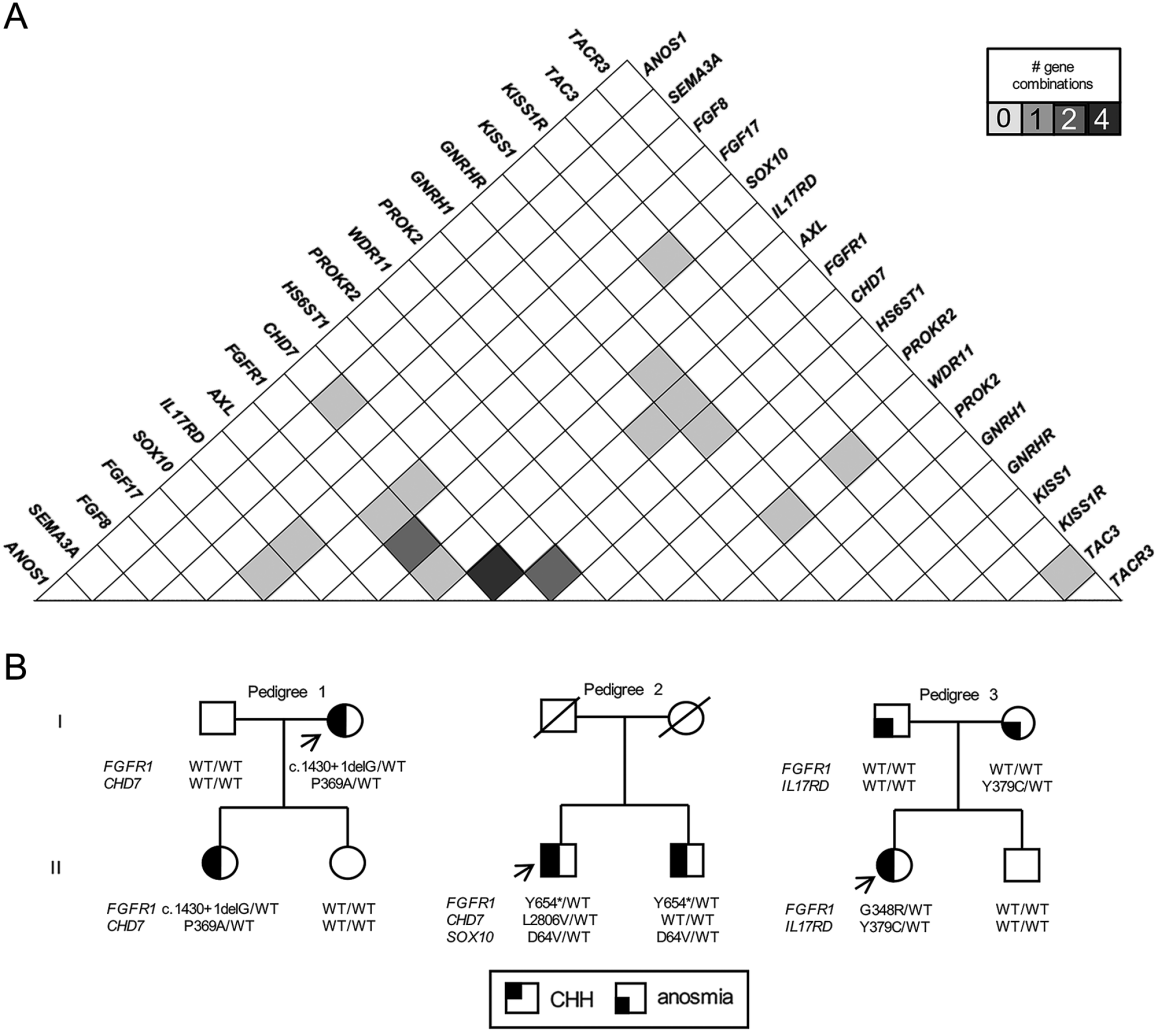
*FGFR1* and *CHD7* are frequently inherited in digenic fashion among CHH probands. (A) Matrix showing shading-coded frequencies CHH genes digenic combinations. (B) Available pedigrees of CHH probands displaying oligogenic inheritance. Circles denote females; squares denote males; arrows depict probands; WT denotes wild-type.

Among possible gene combinations, *FGFR1* and *CHD7* was the most frequent pair interaction (*n* = 4), followed by *FGFR1*/*IL17RD* and *CHD7*/*HS6ST1* (*n* = 2) (Fig. 4A). One KS proband (Fig. 4B, Pedigree 1) carrying mutations in both *CHD7* and *FGFR1*, had two daughters after receiving fertility treatment. One of them carried both mutations and was eventually diagnosed with KS, while the second unaffected daughter did not harbor either of the two changes. In Pedigree 2, we identified three mutated genes (*FGFR1*, *CHD7* and *SOX10*) in a KS proband. His KS brother showed overlapping *FGFR1* and *SOX10* mutations. As there were no phenotypic differences between the proband and his sibling, the *CHD7* mutation is likely not critical in the etiology of KS for this pedigree. Last, we identified a KS proband (Fig. 4B, Pedigree 3) harboring an *IL17RD* mutation inherited from his anosmic mother and a *de novo FGFR1* mutation. We did not identify any CHH gene mutations in the anosmic father or the unaffected brother.

### The majority of mutations in CHH probands are private

When assessing the mutations identified in CHH probands, more than half of them (38/68, 56%) were not found in the ExAC NFE controls (*n* = 33 370), and therefore, are private.

In total, we identified 1492 putative mutations in ExAC NFE controls and 80 mutations in 72/405 (18%) CoLaus controls. However, the majority of mutations in CoLaus (89%, *P* = 6.6 × 10^−4^) (Fig. 2E) occurred in a monoallelic pattern. Given the variant-based (rather than sample-based) nature of the ExAC database, the allelic patterns in these controls could not be assessed.

### Protein-truncating variants are enriched in CHH probands

PTVs are defined as changes predicted to severely disrupt genes, i.e. splicing, frameshift and stop gain variants. A large fraction of the discovered mutations in CHH probands were PTVs (20/68, 29%), while the frequency was significantly lower (5%) in ExAC NFE controls (*P* = 1.0 × 10^−9^). Overall, 18% (*n* = 21) of patients in our cohort harbored at least one PTV in the known CHH genes. Specifically, the CHH cohort was enriched for splice variants in *FGFR1* (2.6%, *P* = 1.7 × 10^−4^) and for frameshift/stop gain variants in *FGFR1* (8%, *P* = 6.9 × 10^−13^), *SOX10* (1.7%, *P* = 1.2 × 10^−5^) and *TACR3* (1.7%, *P* = 4.9 × 10^−3^) when compared to ExAC NFE.

We observed that 80% of PTVs in CHH were in genes with a high constraint to this type of variants (i.e. probability of being loss-of-function intolerant – pLi >0.9) (28), a higher frequency than in ExAC controls (*P* = 0.002). Conversely, the majority of PTVs in ExAC (60%) were present in PTV-tolerant genes (pLi <0.1) (

## Discussion

CDGP and CHH are part of a continuum of GnRH deficiency, ranging from transiently delayed to a complete absence of puberty. However, it is challenging to make a clinical distinction between CHH and CDGP in adolescents presenting with delayed puberty. In this study, we investigated the genetic overlap between these two conditions focusing on rare variants in known CHH genes and *IGSF10*, a gene recently identified in CDGP. CHH and CDGP differ in terms of the number of patients harboring mutations in individual CHH genes (51% vs 7%), as well as the overall mutational load in CHH genes (oligogenicity). In both instances, the CDGP probands more closely resembled the control cohort. We also observed similar frequencies of putative *IGSF10* mutations in CDGP and CHH probands, although higher than previously reported (19). It is important to note, however, that the previous study by Howard and coworkers utilized a different filtering strategy to identify low-frequency variants, specifically focusing on variants with MAF of <2.5% – a level consistent with the frequency of CDGP. Notably, the present study focused on variants with MAF <1.0% given the rarity of the CHH phenotype. Thus, it is not surprising that different results would be achieved. This would suggest that variants with MAF 1.0–2.5% may contribute more strongly to the CDGP phenotype. Indeed, the most frequent *IGSF10* variant in the CDGP cohort (p.Glu161Lys) has a MAF of 2.0% in the Finnish population. In the current study, the variants identified have not been functionally validated nor has segregation with trait within pedigree been analyzed, both of which were used to identify definitive pathogenic variants in the previous study by Howard and coworkers. Furthermore, the lack of an association of *IGSF10* mutations with CDGP or CHH in the current study may reflect a limitation of rare variant burden testing. It is possible that in a very large gene such as *IGSF10*, there may be a large number of non-causal variants or both protective and deleterious variants, and the proportion of these may vary between different populations. In summary, the current data show that the genetic profile of the Finnish CDGP patients, while enriched for rare putative pathogenic variants in *IGSF10* as compared to ethnically matched controls, closely resemble the profile of both ExAC Finnish and non-Finnish control cohorts with respect to known CHH genes.

Recent GWAS studies have identified hundreds of loci associated with puberty onset in the general population (20, 21), with several signals lying close to or within CHH genes suggestive of a genetic overlap between CHH and CDGP. However, GWAS signals may result from intergenic, intronic, promoter or other regulatory changes that are not detected by exome sequencing. Therefore, our results in CHH and CDGP patients could have missed pathogenic mutations in regulatory regions. Notably, a genome-wide significant signal in the coding sequence was reported in *TACR3* (p.Trp275*), a mutation identified in nCHH in this report as well as in previous studies (8, 36, 37). Although prior GWAS studies have not identified an association for its ligand *TAC3*, we identify mutations in *TAC3* in both CHH and CDGP cohorts. Further, *TAC3* mutations were previously reported in CHH as well as CDGP (3). Combined, these data implicate the neurokinin B pathway in both CHH and CDGP. We propose that larger studies examining pathways rather than individual genes identified by GWAS are required to further elucidate the genetic overlap between CHH and CDGP.

Using whole exome sequencing to examine a larger number of CHH genes in our study, we identified mutations in 51% of CHH cases. This is increased in relation to prior reports of 31% (10) and 35% (8) respectively. Our data are mostly consistent with a recent publication by Francou *et al*. (38) that evaluated a large cohort of nCHH patients of European descent for pathogenic variants in *KISS1R*, *GNRHR*, *TACR3*, *KISS1*, *TAC3* and *GNRH1*.

We report a genetic overlap between KS and nCHH. Using a gene-collapsed rare variant association study (RVAS) on the entire CHH cohort, we found significant associations for *FGFR1*, *CHD7* and *SOX10*. Separating CHH into KS and nCHH, the burden test remained significant for *FGFR1* in both subgroups while *CHD7* and *SOX10* were significant only for KS. Notably, significant association appears for *FGF8* in KS while *GNRHR, TACR3* and *KISS1* showed association only in nCHH. A significant enrichment of rare variants in the *RNF216* gene was recently shown in patients with CHH and cerebellar ataxia (39). In contrast, no enrichment in *KISS1* rare variants was detected in 1025 CHH patients, without respect to the phenotypic subgroups (12). These data point toward the importance of phenotypic clustering to identify novel associated genes (8, 40). Finally, our results show that such burden tests might miss associations in important genes like *KISS1R*, because of the low frequency of rare variants in both patient and control population.

Oligogenicity occurs in our study in 15% of CHH cases as compared to 2.5% and 7% in previous reports (8, 10) using nearly identical strategies for variant classification. This increase is due in part to the increased number of CHH genes screened using exome sequencing. Although this study does not provide molecular evidences of oligogenic interactions, previous studies demonstrated that oligogenicity is a critical factor in CHH pathogenesis (8, 11, 41). Recent guidelines from the American College of Medical Genetics aid in the identification of pathogenic variants in a clinical setting (42). While these guidelines are suited only for monogenic disorders, they do provide a structured framework from which to evaluate variants. Using these guidelines, all ACMG pathogenic or likely pathogenic variants were also classified as pathogenic in the current study (Supplementary Table 2). However, a large number of pathogenic variants detected in the current study were classified as unknown significance using ACMG guidelines. This is primarily due to the weight assigned to (i) familial segregation that is not applicable in the setting of oligogenicity and (ii) detection of *de novo* mutations that was not possible in this study as parental DNA was not available for most probands.

The combination of mutations in both *FGFR1* and *CHD7* occurred most frequently (*n* = 4 probands). These two genes might play coordinated roles during GnRH neuron development and migration as CHD7 regulates the transcription of Fgf8, a major ligand for FGFR1 in GnRH neuron ontogeny (11). Moreover, both *FGFR1* and *CHD7* are expressed in relevant tissues for CHH, such as the olfactory bulb and hypothalamus (43). A previous report also suggested functional interactions between these genes, as CHH patients with mutations in *FGFR1* and *CHD7* exhibit overlaps in associated phenotypes (cleft lip/palate, coloboma or ear anomalies) (44).

One notable exception to oligogenicity was *ANOS1* – which was inherited in an exclusively monoallelic fashion due to its X-linked recessive mode of inheritance and high penetrance. Mutations in other genes such as *TAC3*, *KISS1*, *PROK2* and *PROKR2* were primarily biallelic and oligogenic interactions were not observed – likely due to their recessive mode of inheritance. Interestingly, the frequency of monogenic inheritance in KS was significantly higher than in nCHH. To date, it is unclear whether this difference is due to distinct genetic architecture or that ‘missing’ partners in an oligogenic inheritance for KS have yet to be discovered.

We discovered putatively pathogenic mutations in CHH genes in up to 17% of controls, which at first glance seems counterintuitive. Importantly, oligogenicity was only rarely found in controls (2%), further supporting the oligogenic model of CHH pathogenesis. Additionally, many of the putative heterozygous mutations in controls were found in genes with an autosomal recessive inheritance, which would explain the lack of obvious reproductive phenotypes among controls. Further, CHH and controls differ markedly for PTVs (29% vs 5%, respectively), and the PTVs in controls were less likely to be pathogenic.

This study expands our understanding of the genetic architecture of both CHH and CDGP and highlights the very large proportion of cases of CDGP that are not explained by mutations in known genes. Further, the genetic profiles of CHH and CDGP appear to be distinct with respect to the 25 CHH genes studied here, with the understanding that ethnic differences between groups (European vs Finnish) could contribute to this finding. This observation may facilitate differential diagnosis of CHH and CDGP in early adolescence when a clear and early diagnosis is critical to initiate timely induction of puberty in patients with CHH. A genetic test resulting in (1) more than one CHH gene mutated (oligogenicity), (2) hemizygous *ANOS1* mutations in male patients or (3) biallelic mutations in genes associated with autosomal recessive inheritance would favor a diagnosis of CHH. Additional comprehensive studies in larger cohorts may enable genetic testing to inform a more precise differential diagnosis in the clinical setting.

## Supplementary Material

Supporting Figure 1

Supporting Figure 2

Supporting Figure 3

Supporting Figure 4

Supporting Figure 5

Supporting Table 1

Supporting Table 2

Supporting Table 3

## Declaration of interest

The authors declare that there is no conflict of interest that could be perceived as prejudicing the impartiality of this study.

## Funding

This work was supported by the Swiss National Science Foundation grant (SNF 31003A 153328, N P) and by the Shenzhen Municipal Government of China (No. JSGG2015330171719763 and CXB201108250094A). S R H is funded by the Wellcome Trust (102745), Rosetrees Trust (M222) and the Barts and the London Charity (417/1551). L D is partly supported by the Academy of Finland (14135). W S D is supported by an NIHR Research Professorship.

## Author contribution statement

N P and L D designed the research project. D C, J S A, N P, S R H, C X, G P, F S, A S and L D analyzed the data. J M, C C, J L, X L, H Y, J Z prepared and sequenced DNA. J S A and D C managed the project. S R H, C X, S S, L F, L M, P M B, C D G, A D P, W S D, J M F, M H, M L M, J L, C F, A N, F P H, D P, V P, S P, R Q, G S, D A, D K, Sab S, O T I and O D E X team provided for samples DNA and phenotyping. D C, J S A, C X, and N P wrote and prepared the original draft. A A D, G P S, B J S, J S A, N P, C X, L D, S R H reviewed and edited the manuscript.
