# Supplementary figures and images for: Congenital hypogonadotropic hypogonadism and constitutional delay of growth and puberty have distinct genetic architectures
Source: Eur J Endocrinol. 2018 Feb 1;178(4):377–88. doi: 10.1530/EJE-17-0568 (PMC5863472; doi:10.1530/EJE-17-0568)

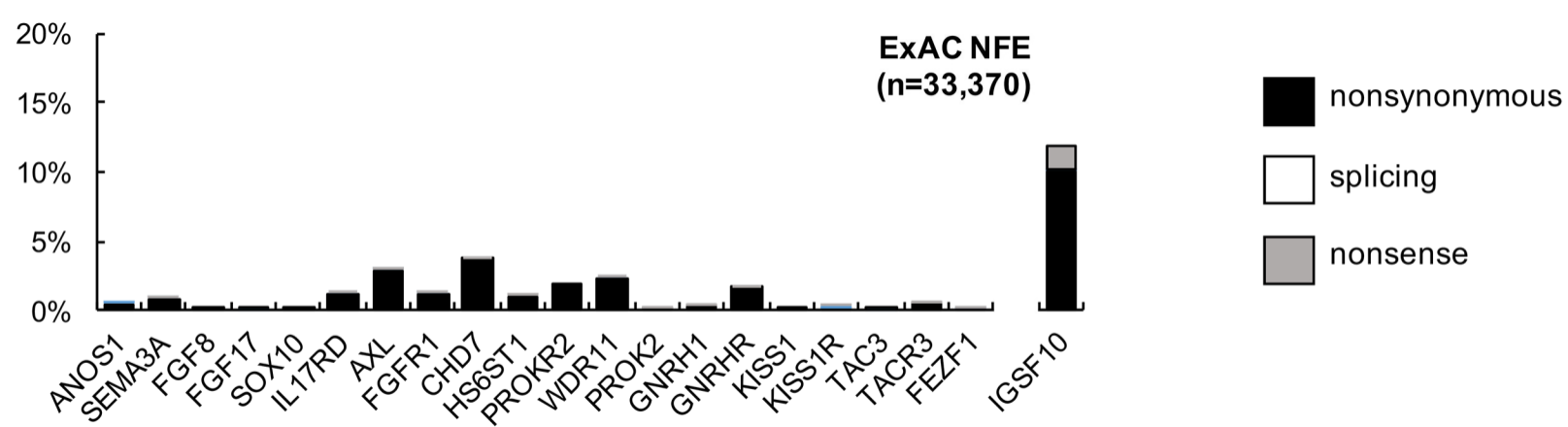

Supplement: Supporting Figure 1 [file eje-178-377-s001.pdf]

## Familial cases

**A**

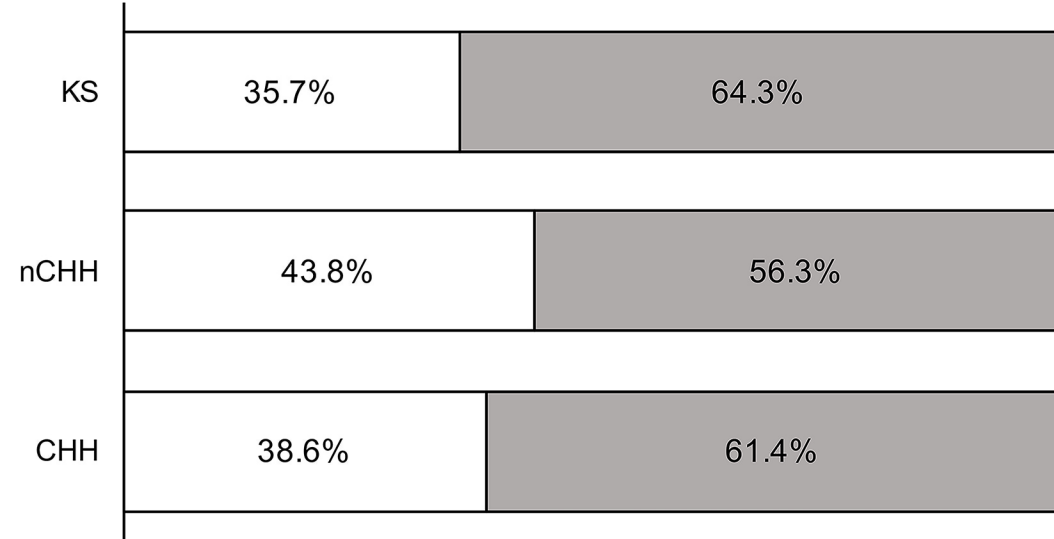

## Sporadic cases

**B**

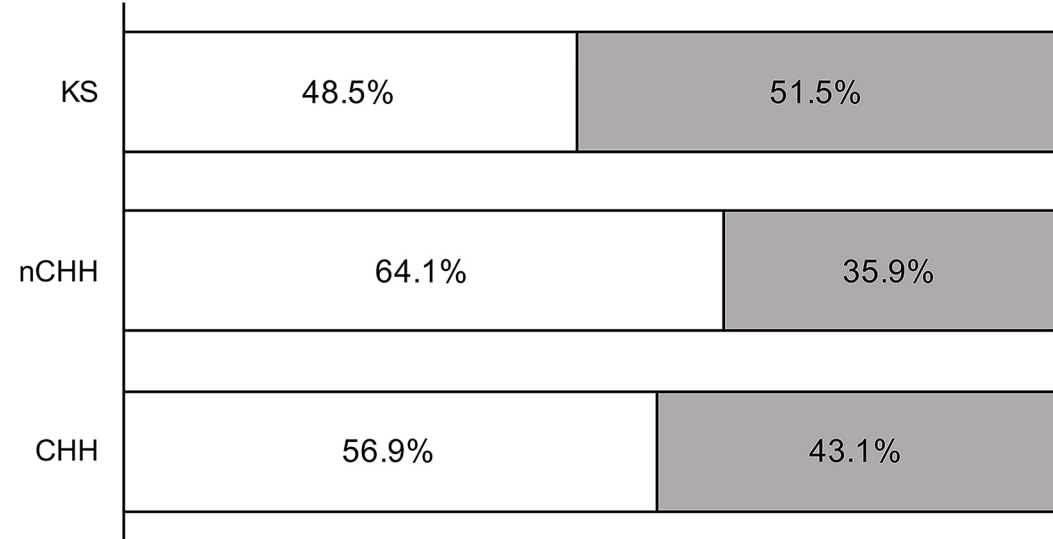

no variants

≥1 gene mutated

Supplement: Supporting Figure 2 [file eje-178-377-s002.pdf]

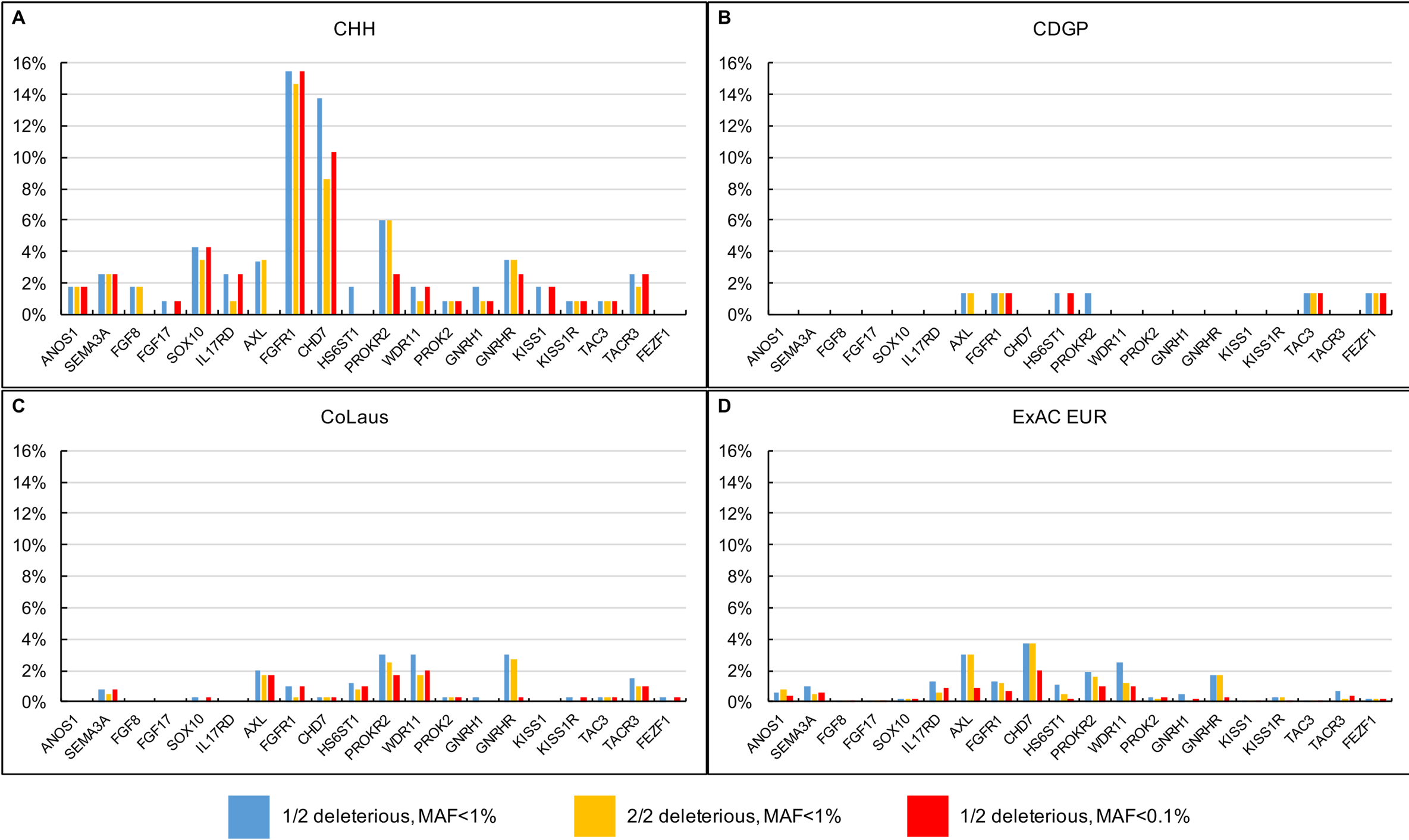

Supplement: Supporting Figure 3 [file eje-178-377-s003.pdf]

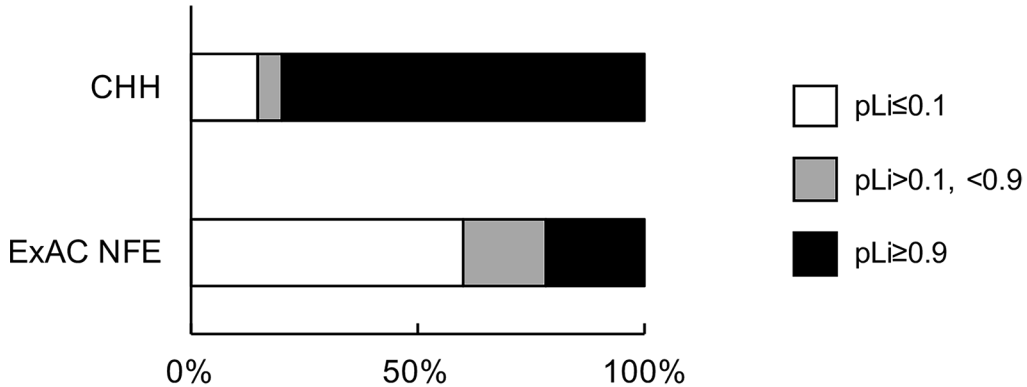

Supplement: Supporting Figure 4 [file eje-178-377-s004.pdf]
