# Supplementary material for: Congenital hypogonadotropic hypogonadism and constitutional delay of growth and puberty have distinct genetic architectures
Source: Eur J Endocrinol. 2018 Feb 1;178(4):377–88. doi: 10.1530/EJE-17-0568 (PMC5863472; doi:10.1530/EJE-17-0568)
Supplement: Supporting Figure 5 [file eje-178-377-s005.pdf]

Supplementary Material

Figure S1. ExAC Non-Finnish European individuals show marginal mutation prevalence in CHH genes. Histograms showing CHH genes mutational estimated prevalence in ExAC non-Finnish European (n=33,370). Each bar contains the frequency of nonsynonymous (i.e., missense and inframe InDels, in black), splicing (in white) and nonsense (i.e., frameshift and stop gained variants, in grey) variants accounting for each gene prevalence. Estimation of prevalence was calculated from the number of heterozygous and homozygous variants found in each gene.

Figure S2. Familial and sporadic cases display different burden of rare variants in CHH genes. Frequencies of familial (A) and sporadic (B) CHH, KS and nCHH probands with no mutations (white) and with at least one mutation in CHH genes (grey).

Figure S3. Different strategies in variants filtering. Mutation prevalence of known CHH genes in CHH (A), CDGP (B), CoLaus (C) and ExAC NFE (D) individuals using three different filtering strategies: (1) MAF<1% in ExAC NFE and at least one deleterious prediction in SIFT and/or PolyPhen-2 for missense variants (blue bars); (2) MAF<1% in ExAC NFE and two deleterious predictions in SIFT and PolyPhen-2 for missense variants (yellow bars); (3) MAF<0.1% in ExAC NFE and at least one deleterious prediction in SIFT and/or PolyPhen-2 for missense variants (red bars).

Figure S4. CHH patients are enriched with PTVs in PTV-intolerant genes. Frequency of PTVs occurring in PTV-intolerant (pLi≥0.9, black) and in PTV-tolerant genes (pLi≤0.1, white) in CHH and ExAC NFE individuals. Intermediate pLi scores (>0.1 and <0.9) are shown in grey.

Table S1. Mutation prevalence of CHH genes in screened cohorts. Prevalence of putative mutations in cases and controls. ExAC NFE prevalence was estimated by dividing the sum of heterozygous and homozygous mutations in each gene to the total population (n=33,370)

Table S2. Putative mutations identified in the CHH cohort. Abbreviations as follows: KS, Kallmann syndrome; nCHH, normosmic congenital hypogonadotropic hypogonadism; Zyg, zygosity; Het, heterozygous; Hom, homozygous; Hem, hemizygous; D, deleterious; T, tolerated; PPH2, PolyPhen-2. PolyPhen-2 “possibly damaging” and “probably damaging” predictions were considered both as “deleterious”, while “benign” were defined as “tolerated” for consistency; LOF, loss-of-function, with experimental data supporting the affected protein functionality with this variant.

Table S3. Number of screened individuals harboring mutated CHH genes. Number and frequency of cases and controls having no rare variants in CHH genes, one gene mutated or at least two genes mutated (oligogenicity). Differences between CHH, KS, and nCHH vs. CDGP probands and controls were analyzed via a two-sided Fisher’s exact test.

Supplementary References

Felix, T.M., Hanshaw, B.C., Mueller, R., Bitoun, P., and Murray, J.C. (2006). CHD7 gene and non-syndromic cleft lip and palate. Am J Med Genet A 140, 2110-2114.

Miraoui, H., Dwyer, A.A., Sykiotis, G.P., Plummer, L., Chung, W., Feng, B., Beenken, A., Clarke, J., Pers, T.H., Dworzynski, P., et al. (2013). Mutations in FGF17, IL17RD, DUSP6, SPRY4, and FLRT3 are identified in individuals with congenital hypogonadotropic hypogonadism. Am J Hum Genet 92, 725-743.

Reynaud, R., Jayakody, S.A., Monnier, C., Saveanu, A., Bouligand, J., Guedj, A.M., Simonin, G., Lecomte, P., Barlier, A., Rondard, P., et al. (2012). PROKR2 variants in multiple hypopituitarism with pituitary stalk interruption. The Journal of clinical endocrinology and metabolism 97, E1068-1073.

Hanchate, N.K., Giacobini, P., Lhuillier, P., Parkash, J., Espy, C., Fouveaut, C., Leroy, C., Baron, S., Campagne, C., Vanacker, C., et al. (2012). SEMA3A, a gene involved in axonal pathfinding, is mutated in patients with Kallmann syndrome. PLoS Genet 8, e1002896.

Balasubramanian, R., Choi, J.H., Francescatto, L., Willer, J., Horton, E.R., Asimacopoulos, E.P., Stankovic, K.M., Plummer, L., Buck, C.L., Quinton, R., et al. (2014). Functionally compromised CHD7 alleles in patients with isolated GnRH deficiency. Proceedings of the National Academy of Sciences of the United States of America 111, 17953-17958.

Pitteloud, N., Meysing, A., Quinton, R., Acierno, J.S., Jr., Dwyer, A.A., Plummer, L., Fliers, E., Boepple, P., Hayes, F., Seminara, S., et al. (2006). Mutations in fibroblast growth factor receptor 1 cause Kallmann syndrome with a wide spectrum of reproductive phenotypes. Molecular and cellular endocrinology 254-255, 60-69.

Guran, T., Tolhurst, G., Bereket, A., Rocha, N., Porter, K., Turan, S., Gribble, F.M., Kotan, L.D., Akcay, T., Atay, Z., et al. (2009). Hypogonadotropic hypogonadism due to a novel missense mutation in the first extracellular loop of the neurokinin B receptor. The Journal of clinical endocrinology and metabolism 94, 3633-3639.

Meysing, A.U., Kanasaki, H., Bedecarrats, G.Y., Acierno, J.S., Jr., Conn, P.M., Martin, K.A., Seminara, S.B., Hall, J.E., Crowley, W.F., Jr., and Kaiser, U.B. (2004). GNRHR mutations in a woman with idiopathic hypogonadotropic hypogonadism highlight the differential sensitivity of luteinizing hormone and follicle-stimulating hormone to gonadotropin-releasing hormone. The Journal of clinical endocrinology and metabolism 89, 3189-3198.

Gianetti, E., Tusset, C., Noel, S.D., Au, M.G., Dwyer, A.A., Hughes, V.A., Abreu, A.P., Carroll, J., Trarbach, E., Silveira, L.F., et al. (2010). TAC3/TACR3 mutations reveal preferential activation of gonadotropin-releasing hormone release by neurokinin B in neonatal life followed by reversal in adulthood. The Journal of clinical endocrinology and metabolism 95, 2857-2867.

Bailleul-Forestier, I., Gros, C., Zenaty, D., Bennaceur, S., Leger, J., and de Roux, N. (2010). Dental agenesis in Kallmann syndrome individuals with FGFR1 mutations. Int J Paediatr Dent 20, 305-312.

Zhu, J., Choa, R.E., Guo, M.H., Plummer, L., Buck, C., Palmert, M.R., Hirschhorn, J.N., Seminara, S.B., and Chan, Y.M. (2015). A shared genetic basis for self-limited delayed puberty and idiopathic hypogonadotropic hypogonadism. The Journal of clinical endocrinology and metabolism 100, E646-654.

Sykiotis, G.P., Plummer, L., Hughes, V.A., Au, M., Durrani, S., Nayak-Young, S., Dwyer, A.A., Quinton, R., Hall, J.E., Gusella, J.F., et al. (2010). Oligogenic basis of isolated gonadotropin-releasing hormone deficiency. Proceedings of the National Academy of Sciences of the United States of America 107, 15140-15144.

Chan, Y.M., Butler, J.P., Pinnell, N.E., Pralong, F.P., Crowley, W.F., Jr., Ren, C., Chan, K.K., and Seminara, S.B. (2011). Kisspeptin resets the hypothalamic GnRH clock in men. The Journal of clinical endocrinology and metabolism 96, E908-915.

Bilan, F., Legendre, M., Charraud, V., Maniere, B., Couet, D., Gilbert-Dussardier, B., and Kitzis, A. (2012). Complete screening of 50 patients with CHARGE syndrome for anomalies in the CHD7 gene using a denaturing high-performance liquid chromatography-based protocol: new guidelines and a proposal for routine diagnosis. J Mol Diagn 14, 46-55.

Cole, L.W., Sidis, Y., Zhang, C., Quinton, R., Plummer, L., Pignatelli, D., Hughes, V.A., Dwyer, A.A., Raivio, T., Hayes, F.J., et al. (2008). Mutations in prokineticin 2 and prokineticin receptor 2 genes in human gonadotrophin-releasing hormone deficiency: molecular genetics and clinical spectrum. The Journal of clinical endocrinology and metabolism 93, 3551-3559.

Falardeau, J., Chung, W.C., Beenken, A., Raivio, T., Plummer, L., Sidis, Y., Jacobson-Dickman, E.E., Eliseenkova, A.V., Ma, J., Dwyer, A., et al. (2008). Decreased FGF8 signaling causes deficiency of gonadotropin-releasing hormone in humans and mice. The Journal of clinical investigation 118, 2822-2831.

Sato, N., Katsumata, N., Kagami, M., Hasegawa, T., Hori, N., Kawakita, S., Minowada, S., Shimotsuka, A., Shishiba, Y., Yokozawa, M., et al. (2004). Clinical assessment and mutation analysis of Kallmann syndrome 1 (KAL1) and fibroblast growth factor receptor 1 (FGFR1, or KAL2) in five families and 18 sporadic patients. The Journal of clinical endocrinology and metabolism 89, 1079-1088.

de Roux, N., Young, J., Misrahi, M., Genet, R., Chanson, P., Schaison, G., and Milgrom, E. (1997). A family with hypogonadotropic hypogonadism and mutations in the gonadotropin-releasing hormone receptor. The New England journal of medicine 337, 1597-1602.

Dode, C., Levilliers, J., Dupont, J.M., De Paepe, A., Le Du, N., Soussi-Yanicostas, N., Coimbra, R.S., Delmaghani, S., Compain-Nouaille, S., Baverel, F., et al. (2003). Loss-of-function mutations in FGFR1 cause autosomal dominant Kallmann syndrome. Nat Genet 33, 463-465.

Raivio, T., Sidis, Y., Plummer, L., Chen, H., Ma, J., Mukherjee, A., Jacobson-Dickman, E., Quinton, R., Van Vliet, G., Lavoie, H., et al. (2009). Impaired fibroblast growth factor receptor 1 signaling as a cause of normosmic idiopathic hypogonadotropic hypogonadism. The Journal of clinical endocrinology and metabolism 94, 4380-4390.

Bergman, J.E., Bocca, G., Hoefsloot, L.H., Meiners, L.C., and van Ravenswaaij-Arts, C.M. (2011). Anosmia predicts hypogonadotropic hypogonadism in CHARGE syndrome. J Pediatr 158, 474-479.

Gonçalves C., Bastos M., Pignatelli D., Borges T., Aragüés J.M., Fonseca F., Pereira B.D., Socorro S., Lemos M.C. (2015) Novel FGFR1 mutations in Kallmann syndrome and normosmic idiopathic hypogonadotropic hypogonadism: evidence for the involvement of an alternatively spliced isoform. Fertil Steril. 2015 Nov;104(5):1261-7
