# Supplementary material for: Congenital hypogonadotropic hypogonadism and constitutional delay of growth and puberty have distinct genetic architectures
Source: Eur J Endocrinol. 2018 Feb 1;178(4):377–88. doi: 10.1530/EJE-17-0568 (PMC5863472; doi:10.1530/EJE-17-0568)
Supplement: Supporting Table 1 [file eje-178-377-t001.pdf]

Table S1. Mutation prevalence of CHH genes in screened cohorts.

Gene	CHH	KS	nCHH	CDGP	CoLaus	1000 Genomes EUR	ExAC NFE
ANOS1	1.7%	3.3%	0.0%	0.0%	0.0%	1.0%	0.6%
SEMA3A	2.6%	4.9%	0.0%	0.0%	0.7%	1.0%	1.0%
FGF8	1.7%	3.3%	0.0%	0.0%	0.0%	0.0%	0.1%
FGF17	0.9%	1.6%	0.0%	0.0%	0.0%	0.0%	0.0%
SOX10	4.3%	6.6%	1.8%	0.0%	0.2%	0.0%	0.2%
IL17RD	2.6%	3.3%	1.8%	0.0%	0.0%	1.5%	1.3%
AXL	3.4%	1.6%	5.5%	1.4%	2.0%	2.0%	3.0%
FGFR1	15.5%	19.7%	10.9%	1.4%	1.0%	0.5%	1.3%
CHD7	13.8%	18.0%	9.1%	0.0%	0.2%	2.5%	3.7%
HS6ST1	1.7%	1.6%	1.8%	1.4%	1.2%	1.0%	1.1%
PROKR2	5.2%	8.2%	1.8%	1.4%	3.0%	2.0%	1.9%
WDR11	1.7%	1.6%	1.8%	0.0%	3.0%	1.5%	2.5%
PROK2	0.9%	0.0%	1.8%	0.0%	0.2%	0.0%	0.2%
GNRH1	1.7%	0.0%	3.6%	0.0%	0.2%	0.0%	0.5%
GNRHR	3.4%	0.0%	7.3%	0.0%	3.0%	0.5%	1.7%
KISS1	1.7%	0.0%	3.6%	0.0%	0.0%	0.0%	0.1%
KISS1R	0.9%	0.0%	1.8%	0.0%	0.2%	0.5%	0.3%
TAC3	0.9%	0.0%	1.8%	1.4%	0.2%	0.0%	0.1%
TACR3	2.6%	0.0%	5.5%	0.0%	1.5%	1.0%	0.7%
FEZF1	0.0%	0.0%	0.0%	1.4%	0.2%	0.5%	0.2%

Prevalence of putative mutations in cases and controls. ExAC NFE prevalence was estimated by dividing the sum of heterozygous and homozygous mutations in each gene to the total population (n=33,370).
