# Supplementary material for: Congenital hypogonadotropic hypogonadism and constitutional delay of growth and puberty have distinct genetic architectures
Source: Eur J Endocrinol. 2018 Feb 1;178(4):377–88. doi: 10.1530/EJE-17-0568 (PMC5863472; doi:10.1530/EJE-17-0568)
Supplement: Supporting Table 2 [file eje-178-377-t002.pdf]

# Table S2. Putative mutations identified in the CHH cohort

| **Sample** | **Phenotype** | **Gene** | **nt change** | **aa change** | **Zyg** | **SIFT** | **PPH2** | **MaxEnt** | ***In vitro*** | **ExAC** | **ExAC NFE** | **Previous report** | **ACMG classification** | **ACMG evidence codes** |
|---|---|---|---|---|---|---|---|---|---|---|---|---|---|---|
| **1** | KS | *CHD7* | c.3056T>G | p.Phe1019Cys | Het | D | D | | | | | | Likely pathogenic | PM1 PM2 PP1 PP3 |
| **2** | KS | *CHD7* | c.1397C>T | p.Ser466Leu | Het | D | T | | | 0.11% | 0.20% | Felix 2006, AJMG | Uncertain significance | BS2 |
| **3** | KS | *CHD7* | c.4914T>G | p.Asp1638Glu | Het | D | D | | | 0.00% | 0.00% | | Uncertain significance | |
| **4** | KS | *FGFR1* | c.2058delC | p.Phe686fs | Het | | | | | | | | Likely pathogenic | PVS1 PM2 |
| **5** | KS | *FGFR1* | c.670G>C | p.Asp224His | Het | D | D | | | | | | Uncertain significance | PM2 PP2 PP3 |
| **6** | KS | *SEMA3A* | c.2201G>A | p.Arg734Gln | Het | D | D | | | 0.00% | 0.00% | | Uncertain significance | PP2 |
| **7** | nCHH | *CHD7* | c.7199G>A | p.Arg2400Gln | Het | T | D | | | 0.00% | 0.00% | | Uncertain significance | BS2 |
| **8** | nCHH | *PROKR2* | c.1019C>G | p.Thr340Ser | Het | D | D | | | | | | Uncertain significance | PM2, PP2 |
| | | | c.332T>G | p.Met111Arg | Het | D | D | | | | | | Uncertain significance | PM1, PM2, PP2 |
| **9** | nCHH | *GNRHR* | c.785G>A | p.Arg262Gln | Hom | D | D | | | 0.20% | 0.24% | De Roux 1997, NEJM | Likely pathogenic | PM1, PM2, PP2, PP3 |
| | | *CHD7* | c.8950C>T | p.Leu2984Phe | Het | D | T | | | 0.45% | 0.97% | | Likely benign | BS2 BP6 |

| | | | | | | | | | | | | | | |
|---|---|---|---|---|---|---|---|---|---|---|---|---|---|---|
| **10** | KS | *ANOS1* | c.1756C>T | p.Gln586* | Hem | | | | | | | Miraoui 2013, AJHG | Pathogenic | PVS1, PM2, PP3 |
| **11** | KS | *SOX10* | c.267delC | p.Met90fs | Het | | | | | | | | Likely pathogenic | PVS1, PM2 |
| **12** | KS | *PROKR 2* | c.518T>G | p.Leu173Arg | Het | D | D | | LOF | 0.22% | 0.35% | Reynaud 2012, JCEM | Likely pathogenic | PS3, PM1, PP2, PP5, BS2 |
| **13** | KS | *SEMA3A* | c.196C>T | p.Arg66Trp | Het | D | D | | | 0.05% | 0.08% | Hanchate 2012, Plos Genet | Likely pathogenic | PS3, PM1, PP2 |
| | | *CHD7* | c.4847A>G | p.Tyr1616Cys | Het | D | D | | | | | Balasubramanian 2014, PNAS | Uncertain significance | PM2 PP3 |
| **14** | KS | *IL17RD* | c.1690T>G | p.Phe564Val | Het | T | D | | | 0.01% | 0.01% | | Uncertain significance | PM1, BS4 |
| | | *FGFR1* | c.232C>T | p.Arg78Cys | Het | D | D | | | | | Pitteloud 2006, Mol Cell Endocrinol | Pathogenic | PS3 PM1 PM2 PP1 PP2 PP3 |
| **15** | KS | *FGF17* | c.287G>A | p.Arg96Gln | Het | T | D | | | | | | Uncertain significance | PP1, PP4 |
| **16** | KS | *FGFR1* | c.1430+1delG | | Het | | | -207.8% | | | | | Pathogenic | PVS1 PM2 PP1 |
| | | *CHD7* | c.1105C>G | p.Pro369Ala | Het | T | D | | | 0.00% | 0.00% | | Uncertain significance | PP1 |
| **17** | KS | *PROKR 2* | c.518T>G | p.Leu173Arg | Het | D | D | | LOF | 0.22% | 0.35% | Reynaud 2012, JCEM | Likely pathogenic | PS3, PM1, PP2, PP5, BS2 |
| **18** | nCHH | *AXL* | c.1549G>A | p.Gly517Ser | Het | D | D | | | 0.45% | 0.68% | | Uncertain significance | PS2, PP3, BS4 |
| **19** | nCHH | *GNRH1* | c.87delA | p.Leu30fs | Hom | | | | | | | | Likely pathogenic | PVS1 PM2 |
| **20** | nCHH | *TACR3* | c.443A>T | p.His148Leu | Hom | D | D | | LOF | | | Guran 2009, JCEM | Pathogenic | PS3, PM1, PM2, PM3, PP1 |
| **21** | nCHH | *FGFR1* | c.1756_1763dupAACCCCAG | p.Ser588fs | Het | | | | | | | | Likely pathogenic | PVS1 PM2 |
| **22** | KS | *SEMA3A* | c.1360+2T>G | | Het | | | -87.2% | | | | | Likely pathogenic | PVS1, PM2, BS4 |
| **23** | KS | *IL17RD* | c.1136A>G | p.Tyr379Cys | Het | T | D | | LOF | 0.01% | 0.01% | Miraoui 2013, AJHG | Likely pathogenic | PM1, PS3 |
| | | *FGFR1* | c.1042G>A | p.Gly348Arg | Het | D | D | | | | | | Likely pathogenic | PS2 PM2 PP2 PP3 |

| | | | | | | | | | | | | | |
|---|---|---|---|---|---|---|---|---|---|---|---|---|---|
| **24** | KS | *WDR11* | c.1342C>T | p.Arg448Trp | Het | D | D | | 0.01% | 0.00% | | Uncertain significance | PP2, PP3 |
| **25** | nCHH | *GNRHR* | c.31C>A | p.Gln11Lys | Het | T | T | LOF | 0.02% | 0.03% | Meysing 2004, JCEM | Likely pathogenic | PSA3, PM2, PP2 |
| | | | c.30T>A | p.Asn10Lys | Het | T | T | LOF | 0.02% | 0.03% | Gianetti 2010, JCEM | Likely pathogenic | PS3, PM2, PP2, PP5, BP4 |
| | | *WDR11* | c.1342C>T | p.Arg448Trp | Het | D | D | | 0.01% | 0.00% | | Uncertain significance | PP2, PP3 |
| **26** | nCHH | *TACR3* | c.824G>A | p.Trp275* | Ho m | | | | 0.02% | 0.04% | Gianetti 2010, JCEM | Pathogenic | PVS1, PS3, PM2, PP1, PP3, PP5 |
| **27** | nCHH | *KISS1R* | c.772C>T | p.Arg258Trp | Het | D | D | | 0.00% | 0.00% | | Uncertain significance | PM1, PM2, PP3 |
| | | *SOX10* | c.481C>T | p.Arg161Cys | Het | D | D | | | | | Uncertain significance | PM1, PM2, PP3 |
| **28** | KS | *FGFR1* | c.1042G>A | p.Gly348Arg | Het | D | D | | | | Bailleul-Forestier 2010, Int J Paediatr Dent | Likely pathogenic | PS2 PM2 PP2 PP3 |
| **29** | KS | *AXL* | c.1549G>A | p.Gly517Ser | Het | D | D | | 0.45% | 0.68% | | Uncertain significance | PS2, PP3, BS4 |
| **30** | KS | *PROKR 2* | c.518T>G | p.Leu173Arg | Het | D | D | LOF | 0.22% | 0.35% | Reynaud 2012, JCEM | Likely pathogenic | PS3, PM1, PP2, PP5, BS2 |
| **31** | nCHH | *GNRHR* | c.350T>G | p.Leu117Arg | Het | D | D | | | | Zhu 2015, JCEM | Likely pathogenic | PM1, PM2, PP2, PP5 |
| | | | c.266T>A | p.Leu89* | Het | | | | | | | Pathogenic | PM2, PP3, PM3, PVS1 |
| | | *FGFR1* | c.1368G>T | p.Met456Ile | Het | D | T | | 0.04% | 0.07% | Sykiotis 2010, PNAS | Uncertain significance | PM1 PM2 PP2 |
| | | *AXL* | c.1549G>A | p.Gly517Ser | Het | D | D | | 0.45% | 0.68% | | Uncertain significance | PS2, PP3, BS4 |

| | | | | | | | | | | | | | | |
|---|---|---|---|---|---|---|---|---|---|---|---|---|---|---|
| **32** | nCHH | *KISS1* | c.349T>C | p.Phe117Leu | Het | T | T | | LOF | 0.02% | 0.07% | Chan 2011, JCEM | Uncertain significance | PM2, PP2, PP3 |
| **33** | nCHH | *KISS1* | c.154_156dupCCG | p.Pro52dup | Het | | | | | 0.00% | 0.00% | | Uncertain significance | PM2 |
| **34** | KS | *ANOS1* | c.256T>A | p.Cys86Ser | Hem | D | D | | | | | | Uncertain significance | PM2, PP3 |
| **35** | KS | *FGFR1* | c.1961dupA | p.Tyr654* | Het | | | | | | | Goncalves 2014, Fertil Steril | Pathogenic | PVS1 PM2 PP1 |
| | | *CHD7* | c.8416C>G | p.Leu2806Val | Het | T | D | | | 0.11% | 0.09% | Bilan 2012, J Mol Diag | Benign | BS1 BS2 BS4 |
| | | *SOX10* | c.191A>T | p.Asp64Val | Het | D | T | | | 0.00% | 0.00% | | Uncertain significance | PM1, PP1, PP3 |
| **36** | nCHH | *PROK2* | c.163delA | p.Ile55fs | Hom | | | | | 0.01% | 0.02% | Cole 2008, JCEM | Pathogenic | PVS1, PS3 |
| **37** | KS | *FGF8* | c.77C>T | p.Pro26Leu | Het | T | T | | LOF | 0.04% | 0.49% | Falardeau 2008, JCI | Likely pathogenic | PS3, PP2, PP5, BP4 |
| | | *SOX10* | c.89C>A | p.Ser30* | Het | | | | | | | | Pathogenic | PVS1, PM2, PP3 |
| **38** | KS | *PROKR2* | c.271C>T | p.Leu91Phe | Het | D | D | | | 0.00% | 0.00% | | Uncertain significance | PM1, PP2 |
| **39** | nCHH | *HS6ST1* | c.652C>T | p.Pro218Ser | Het | T | D | | | 0.16% | 0.27% | | Uncertain significance | PM2, PP2 |
| | | *CHD7* | c.5051-4C>T | | Het | | | +20.3% | | 0.54% | 0.80% | | Uncertain significance | |
| **40** | nCHH | *GNRH1* | c.141G>C | p.Glu47Asp | Het | T | D | | | 0.14% | 0.17% | | Benign | PM2 PP3 BS2 BS4 |
| | | *FGFR1* | c.622-1G>T | | Het | | | -134.5% | | | | | Pathogenic | PVS1 PM2 PP1 |
| **41** | nCHH | *AXL* | c.1549G>A | p.Gly517Ser | Het | D | D | | | 0.45% | 0.68% | | Benign | PS2, PP3, BS1, BS4 |
| **42** | KS | *FGFR1* | c.2233C>T | p.Pro745Ser | Het | D | D | | | | | Sato 2004, JCEM | Uncertain significance | PM2 PP2 PP3 |
| **43** | nCHH | *FGFR1* | c.2464C>T | p.Arg822Cys | Het | D | D | | | 0.02% | 0.02% | | Uncertain significance | PM2 PP2 PP3 |
| **44** | nCHH | *TACR3* | c.824G>A | p.Trp275* | Het | | | | | 0.02% | 0.04% | Gianetti 2010, JCEM | Pathogenic | PVS1, PS3, PM2, PP1, PP3, PP5 |
| | | *TAC3* | c.248A>G | p.His83Arg | Het | D | D | | | 0.02% | 0.02% | | Uncertain | PM2, PP3, PP5 |

| | | | | | | | | | | | | | | |
|---|---|---|---|---|---|---|---|---|---|---|---|---|---|---|
| | | | | | | | | | | | | | significance | |
| **45** | KS | *FGFR1* | c.1093_1094dupAG | p.Pro366fs | Het | | | | | | | | Likely pathogenic | PVS1 PM2 |
| | | *CHD7* | c.8188G>A | p.Ala2730Thr | Het | T | D | | | 0.00% | 0.00% | | Uncertain significance | PP1 |
| **46** | nCHH | *IL17RD* | c.2068T>A | p.Ser690Thr | Het | D | D | | | 0.00% | 0.01% | | Uncertain significance | |
| | | *GNRHR* | c.784C>T | p.Arg262Trp | Het | D | D | | LOF | 0.00% | 0.00% | | Likely pathogenic | PM1, PM2, PP2, PP3 |
| | | | c.317A>G | p.Gln106Arg | Het | D | D | | LOF | 0.25% | 0.40% | De Roux 1997, NEJM | Pathogenic | PM1, PM2, PP2, PP5, BS2 |
| **47** | KS | *FGFR1* | c.1038_1039insTT | p.Ile347fs | Het | | | | | | | | Pathogenic | PVS1 PM2 PP1 |
| **48** | KS | *CHD7* | c.7282C>T | p.Arg2428* | Het | | | | | | | | Pathogenic | PVS1 PM2 PM6 |
| **49** | KS | *FGFR1* | c.296A>G | p.Tyr99Cys | Het | D | D | | LOF | | | Dodè 2003, Nat Genet; Raivio 2009, JCEM | Likely pathogenic | PS3 PM2 PP2 PP3 PP5 |
| **50** | KS | *PROKR 2* | c.518T>G | p.Leu173Arg | Het | D | D | | LOF | 0.22% | 0.35% | Reynaud 2012, JCEM | Likely pathogenic | PS3, PM1, PP2, PP5, BS2 |
| **51** | KS | *HS6ST1* | c.652C>T | p.Pro218Ser | Het | T | D | | | 0.16% | 0.27% | | Uncertain significance | PM2, PP2 |
| | | *CHD7* | c.2966G>A | p.Cys989Tyr | Het | D | D | | | | | | Likely pathogenic | PM1 PM2 PM6 PP3 |
| **52** | KS | *FGFR1* | c.790A>T | p.Asn264Tyr | Het | D | D | | | | | | Likely pathogenic | PM1 PM2 PP2 PP3 |
| **53** | KS | *CHD7* | c.5051-4C>T | | Het | | | +20.3% | | 0.54% | 0.80% | | Uncertain significance | |
| **54** | nCHH | *FGFR1* | c.1306_1307dupTC | p.Met437fs | Het | | | | | | | | Pathogenic | PVS1 PM2 PP1 |
| | | *CHD7* | c.2613+5G>A | | Het | | | -40.7% | | 0.00% | 0.00% | | Uncertain significance | PM1 PP1 |

| | | | | | | | | | | | | | | |
|---|---|---|---|---|---|---|---|---|---|---|---|---|---|---|
| **55** | KS | *SOX10* | c.530G>A | p.Arg177Gln | Het | D | D | | | | | | Uncertain significance | PM1, PM2, PP3 |
| **56** | KS | *CHD7* | c.3320C>T | p.Ala1107Val | Het | D | D | | | | | | Likely pathogenic | PM1 PM2 PP1 PP3 |
| **57** | nCHH | *CHD7* | c.3973T>C | p.Tyr1325His | Het | D | D | | | 0.00% | 0.01% | Bergman 2011, J Pediatr | Uncertain significance | PM1 PP3 |
| **58** | nCHH | *FGFR1* | c.1552+1G>A | | Het | | | -96.8% | | | | | Pathogenic | PVS1 PM2 PP1 |
| **59** | KS | *FGF8* | c.77C>T | p.Pro26Leu | Het | T | T | | LOF | 0.04% | 0.49% | Falardeau 2008, JCI | Uncertain significance | PP2 PP5 BP4 |

Abbreviations as follows: KS, Kallmann syndrome; nCHH, normosmic congenital hypogonadotropic hypogonadism; Zyg, zygosity; Het, heterozygous; Hom, homozygous; Hem, hemizygous; D, deleterious; T, tolerated; PPH2, PolyPhen-2. PolyPhen-2 "possibly damaging" and "probably damaging" predictions were considered both as "deleterious", while "benign" were defined as "tolerated" for consistency; LOF, loss-of-function, with experimental data supporting the affected protein functionality with this variant.

## Supplementary References


Felix, T.M., Hanshaw, B.C., Mueller, R., Bitoun, P., and Murray, J.C. (2006). CHD7 gene and non-syndromic cleft lip and palate. Am J Med Genet A 140, 2110-2114.

Miraoui, H., Dwyer, A.A., Sykiotis, G.P., Plummer, L., Chung, W., Feng, B., Beenken, A., Clarke, J., Pers, T.H., Dworzynski, P., et al. (2013). Mutations in FGF17, IL17RD, DUSP6, SPRY4, and FLRT3 are identified in individuals with congenital hypogonadotropic hypogonadism. Am J Hum Genet 92, 725-743.

Reynaud, R., Jayakody, S.A., Monnier, C., Saveanu, A., Bouligand, J., Guedj, A.M., Simonin, G., Lecomte, P., Barlier, A., Rondard, P., et al. (2012). PROKR2 variants in multiple hypopituitarism with pituitary stalk interruption. The Journal of clinical endocrinology and metabolism 97, E1068-1073.

Hanchate, N.K., Giacobini, P., Lhuillier, P., Parkash, J., Espy, C., Fouveaut, C., Leroy, C., Baron, S., Campagne, C., Vanacker, C., et al. (2012). SEMA3A, a gene involved in axonal pathfinding, is mutated in patients with Kallmann syndrome. PLoS Genet 8, e1002896.

Balasubramanian, R., Choi, J.H., Francescatto, L., Willer, J., Horton, E.R., Asimacopoulos, E.P., Stankovic, K.M., Plummer, L., Buck, C.L., Quinton, R., et al. (2014). Functionally compromised CHD7 alleles in patients with isolated GnRH deficiency. Proceedings of the National Academy of Sciences of the United States of America 111, 17953-17958.

Pitteloud, N., Meysing, A., Quinton, R., Acierno, J.S., Jr., Dwyer, A.A., Plummer, L., Fliers, E., Boepple, P., Hayes, F., Seminara, S., et al. (2006). Mutations in fibroblast growth factor receptor 1 cause Kallmann syndrome with a wide spectrum of reproductive phenotypes. Molecular and cellular endocrinology 254-255, 60-69.

Guran, T., Tolhurst, G., Bereket, A., Rocha, N., Porter, K., Turan, S., Gribble, F.M., Kotan, L.D., Akcay, T., Atay, Z., et al. (2009). Hypogonadotropic hypogonadism due to a novel missense mutation in the first extracellular loop of the neurokinin B receptor. The Journal of clinical endocrinology and metabolism 94, 3633-3639.

Meysing, A.U., Kanasaki, H., Bedecarrats, G.Y., Acierno, J.S., Jr., Conn, P.M., Martin, K.A., Seminara, S.B., Hall, J.E., Crowley, W.F., Jr., and Kaiser, U.B. (2004). GNRHR mutations in a woman with idiopathic hypogonadotropic hypogonadism highlight the differential sensitivity of luteinizing hormone and follicle-stimulating hormone to gonadotropin-releasing hormone. The Journal of clinical endocrinology and metabolism 89, 3189-3198.

Gianetti, E., Tusset, C., Noel, S.D., Au, M.G., Dwyer, A.A., Hughes, V.A., Abreu, A.P., Carroll, J., Trarbach, E., Silveira, L.F., et al. (2010). TAC3/TACR3 mutations reveal preferential activation of gonadotropin-releasing hormone release by neurokinin B in neonatal life followed by reversal in adulthood. The Journal of clinical endocrinology and metabolism 95, 2857-2867.

Bailleul-Forestier, I., Gros, C., Zenaty, D., Bennaceur, S., Leger, J., and de Roux, N. (2010). Dental agenesis in Kallmann syndrome individuals with FGFR1 mutations. Int J Paediatr Dent 20, 305-312.

Zhu, J., Choa, R.E., Guo, M.H., Plummer, L., Buck, C., Palmert, M.R., Hirschhorn, J.N., Seminara, S.B., and Chan, Y.M. (2015). A shared genetic basis for self-limited delayed puberty and idiopathic hypogonadotropic hypogonadism. The Journal of clinical endocrinology and metabolism 100, E646-654.

Sykiotis, G.P., Plummer, L., Hughes, V.A., Au, M., Durrani, S., Nayak-Young, S., Dwyer, A.A., Quinton, R., Hall, J.E., Gusella, J.F., et al. (2010). Oligogenic basis of isolated gonadotropin-releasing hormone deficiency. Proceedings of the National Academy of Sciences of the United States of America 107, 15140-15144.

Chan, Y.M., Butler, J.P., Pinnell, N.E., Pralong, F.P., Crowley, W.F., Jr., Ren, C., Chan, K.K., and Seminara, S.B. (2011). Kisspeptin resets the hypothalamic GnRH clock in men. The Journal of clinical endocrinology and metabolism 96, E908-915.

Bilan, F., Legendre, M., Charraud, V., Maniere, B., Couet, D., Gilbert-Dussardier, B., and Kitzis, A. (2012). Complete screening of 50 patients with CHARGE syndrome for anomalies in the CHD7 gene using a denaturing high-performance liquid chromatography-based protocol: new guidelines and a proposal for routine diagnosis. J Mol Diagn 14, 46-55.

Cole, L.W., Sidis, Y., Zhang, C., Quinton, R., Plummer, L., Pignatelli, D., Hughes, V.A., Dwyer, A.A., Raivio, T., Hayes, F.J., et al. (2008). Mutations in prokineticin 2 and prokineticin receptor 2 genes in human gonadotrophin-releasing hormone deficiency: molecular genetics and clinical spectrum. The Journal of clinical endocrinology and metabolism 93, 3551-3559.

Falardeau, J., Chung, W.C., Beenken, A., Raivio, T., Plummer, L., Sidis, Y., Jacobson-Dickman, E.E., Eliseenkova, A.V., Ma, J., Dwyer, A., et al. (2008). Decreased FGF8 signaling causes deficiency of gonadotropin-releasing hormone in humans and mice. The Journal of clinical investigation 118, 2822-2831.

Sato, N., Katsumata, N., Kagami, M., Hasegawa, T., Hori, N., Kawakita, S., Minowada, S., Shimotsuka, A., Shishiba, Y., Yokozawa, M., et al. (2004). Clinical assessment and mutation analysis of Kallmann syndrome 1 (KAL1) and fibroblast growth factor receptor 1 (FGFR1, or KAL2) in five families and 18 sporadic patients. The Journal of clinical endocrinology and metabolism 89, 1079-1088.

de Roux, N., Young, J., Misrahi, M., Genet, R., Chanson, P., Schaison, G., and Milgrom, E. (1997). A family with hypogonadotropic hypogonadism and mutations in the gonadotropin-releasing hormone receptor. The New England journal of medicine 337, 1597-1602.

Dode, C., Levilliers, J., Dupont, J.M., De Paepe, A., Le Du, N., Soussi-Yanicostas, N., Coimbra, R.S., Delmaghani, S., Compain-Nouaille, S., Baverel, F., et al. (2003). Loss-of-function mutations in FGFR1 cause autosomal dominant Kallmann syndrome. Nat Genet 33, 463-465.

Raivio, T., Sidis, Y., Plummer, L., Chen, H., Ma, J., Mukherjee, A., Jacobson-Dickman, E., Quinton, R., Van Vliet, G., Lavoie, H., et al. (2009). Impaired fibroblast growth factor receptor 1 signaling as a cause of normosmic idiopathic hypogonadotropic hypogonadism. The Journal of clinical endocrinology and metabolism 94, 4380-4390.

Bergman, J.E., Bocca, G., Hoefsloot, L.H., Meiners, L.C., and van Ravenswaaij-Arts, C.M. (2011). Anosmia predicts hypogonadotropic hypogonadism in CHARGE syndrome. J Pediatr 158, 474-479.

Gonçalves C., Bastos M., Pignatelli D., Borges T., Aragüés J.M., Fonseca F., Pereira B.D., Socorro S., Lemos M.C. (2015) Novel FGFR1 mutations in Kallmann syndrome and normosmic idiopathic hypogonadotropic hypogonadism: evidence for the involvement of an alternatively spliced isoform. Fertil Steril. 2015 Nov;104(5):1261-7
