# Supplementary material for: Congenital hypogonadotropic hypogonadism and constitutional delay of growth and puberty have distinct genetic architectures
Source: Eur J Endocrinol. 2018 Feb 1;178(4):377–88. doi: 10.1530/EJE-17-0568 (PMC5863472; doi:10.1530/EJE-17-0568)
Supplement: Supporting Table 3 [file eje-178-377-t003.pdf]

Table S3. Number of screened individuals harboring mutated CHH genes.

	CHH	% CHH	p-value vs. CoLaus	p-value vs. 1000G	p-value vs. CDGP	KS	% KS	p-value vs. CoLaus	p-value vs. 1000G	p-value vs. CDGP	nCHH	% nCHH	p-value vs. CoLaus	p-value vs. 1000G	p-value vs. CDGP	CDGP	% CDGP	CoLaus	% CoLaus	1000G	% 1000G
**0 genes**	57	50.0%	5.5E-12	6.9E-14	7.7E-11	25	42.6%	6.8E-11	5.6E-13	5.4E-11	32	58.2%	1.3E-04	2.0E-06	3.0E-06	67	93.1%	333	82.2%	174	88.3%
**1 gene**	42	35.3%	5.8E-06	1.5E-07	5.8E-07	28	44.3%	4.6E-07	1.2E-08	3.7E-08	14	25.5%	ns	0.008	0.002	4	5.6%	64	15.8%	21	10.7%
**≥2 genes**	17	14.7%	6.4E-07	1.7E-06	0.002	8	13.1%	1.4E-05	2.0E-04	0.012	9	16.4%	3.0E-05	2.5E-05	0.002	1	1.4%	8	2.0%	2	1.0%

Number and frequency of cases and controls having no rare variants in CHH genes, one gene mutated or at least two genes mutated (oligogenicity). Differences between CHH, KS, and nCHH vs. CDGP probands and controls were analyzed via a two-sided Fisher's exact test.
